# Supplementary material for: FERN – a Java framework for stochastic simulation and evaluation of reaction networks
Source: BMC Bioinformatics. 2008 Aug 29;9:356. doi: 10.1186/1471-2105-9-356 (PMC2553347; doi:10.1186/1471-2105-9-356)
Supplement: Additional file 1 — FERN distribution, Version 1.3. This archive contains the FERN source code and binaries as well as documentation and example models in FernML and SBML. [file 1471-2105-9-356-S1.zip › fern/doc/javadoc/index-files/index-3.html]

C-Index


---


|  |  |  |
| --- | --- | --- |
| **Overview** \| Package \| Class \| Use \| **Tree** \| **Deprecated** \| **Index** \| **Help** | |  |
| **PREV LETTER**   **NEXT LETTER** | **FRAMES**    **NO FRAMES**     **All Classes** |  |


A B C D E F G H I J L M N O P R S T U V 

---


## **C**

**calculate(AmountManager, Simulator)** - Method in class fern.network.sbml.MathTree: Evaluate the MathTree. **calculateHistogramDistance(Map<Integer, Integer>, Map<Integer, Integer>)** - Static method in class fern.tools.NumberTools: Calculates the histogram distance of two histograms. **calculateNodeAppearance(NodeAppearance, Node, CyNetwork)** - Method in class fern.cytoscape.CytoscapeColorChangeObserver.ColorChangingNodeAppeareanceCalculator: **calculatePartialDerivative(int, AmountManager, int, double)** - Method in class fern.network.AbstractKineticConstantPropensityCalculator: Calculates partial differentials of the propensity functions for the tau leaping methods. **calculatePropensity(int, AmountManager, Simulator)** - Method in class fern.cellDesigner.CellDesignerPropensityCalculator: **calculatePropensity(int, AmountManager, Simulator)** - Method in class fern.network.AbstractKineticConstantPropensityCalculator: Calculates the propensity for `reaction` by the formula h\*c, where c is the kinetic constant for `reaction` and h is the number of distinct molecular reactant combinations for `reaction`. **calculatePropensity(int, AmountManager, Simulator)** - Method in interface fern.network.PropensityCalculator: Calculates the propensity for a reaction given the amounts of the `AmountManager`. **calculatePropensity(int, AmountManager, Simulator)** - Method in class fern.network.sbml.SBMLPropensityCalculator: **CatalysedNetwork** - Class in fern.network.modification: Modifies the network by adding reactions X+C -> Y+C (where C is each catalyst of the original reaction). **CatalysedNetwork(Network)** - Constructor for class fern.network.modification.CatalysedNetwork: Create a catalyzed network from an original network. **CatalystIterator** - Interface in fern.network.creation: A `CatalystIterator` is used to enumerate the catalysts of a reaction in an `AutocatalyticNetwork`. **CATALYSTS\_FIELD** - Static variable in class fern.network.creation.AutocatalyticNetwork: Name of the field where catalysts are stored. **CATALYSTS\_FIELD\_REVERSIBLE** - Static variable in class fern.network.creation.AutocatalyticNetwork: Name of the field where catalysts for the reverse reactions are stored. **CellDesignerNetworkWrapper** - Class in fern.cellDesigner: **CellDesignerNetworkWrapper(PluginModel)** - Constructor for class fern.cellDesigner.CellDesignerNetworkWrapper: **CellDesignerPropensityCalculator** - Class in fern.cellDesigner: Propensity calculator which is used for `SBMLNetwork`s. **CellDesignerPropensityCalculator(PluginModel, CellDesignerNetworkWrapper)** - Constructor for class fern.cellDesigner.CellDesignerPropensityCalculator: Creates the `MathTree`s and parses the parameters. **CellGrowthObserver** - Class in fern.example: Does not observe anything, but it controls the reaction networks cell. **CellGrowthObserver(Simulator, double, double)** - Constructor for class fern.example.CellGrowthObserver: Creates the observer for given simulator, generation time and a time offset (because the simulator starts at time 0 and for one example it actually starts at time 1000) **check()** - Method in class fern.cytoscape.NetworkChecker: **checkMatrix()** - Method in class fern.tools.gnuplot.ArrayMatrixAxes: Checks if the passed object is a valid array matrix. **checkReaction(int, NetworkSearchAction.NeighborType)** - Method in interface fern.analysis.NetworkSearchAction: Gets called, before the reaction is inserted into the search structure. **checkReactionNode(Network, int)** - Method in interface fern.analysis.NodeChecker: **checkReactionNode(Network, int)** - Method in class fern.analysis.NodeCheckerByAnnotation: **checkSpecies(int, NetworkSearchAction.NeighborType)** - Method in interface fern.analysis.NetworkSearchAction: Gets called, before the species is inserted into the search structure. **checkSpeciesNode(Network, int)** - Method in interface fern.analysis.NodeChecker: **checkSpeciesNode(Network, int)** - Method in class fern.analysis.NodeCheckerByAnnotation: **Children** - Variable in class fern.network.sbml.MathTree.InnerNode: **chooseTauCriticals(BitVector)** - Method in class fern.simulation.algorithm.AbstractBaseTauLeaping: choose the timestep candidate for the critical reactions **chooseTauNonCriticals(BitVector)** - Method in class fern.simulation.algorithm.AbstractBaseTauLeaping: choose the timestep candidate for the noncritical reactions by some method. **chooseTauNonCriticals(BitVector)** - Method in class fern.simulation.algorithm.AbstractTauLeapingPropensityBoundSimulator: **chooseTauNonCriticals(BitVector)** - Method in class fern.simulation.algorithm.TauLeapingSpeciesPopulationBoundSimulator: **clearData()** - Method in class fern.benchmark.Benchmark: Clears all collected benchmark data. **clearData()** - Method in class fern.tools.gnuplot.GnuPlot: Clears the loaded data. **clearedAxes** - Variable in class fern.tools.gnuplot.GnuPlot: **clone()** - Method in class fern.cytoscape.ColorCalculator: **coefficient** - Variable in class fern.cytoscape.NetworkChecker: **CollectionAxes** - Class in fern.tools.gnuplot: Implementation of `Axes` to use with `Collection`s or `Iterable`s of any number type or array of numbers (the type of the collection is inferred by using the reflection api). **CollectionAxes(Collection)** - Constructor for class fern.tools.gnuplot.CollectionAxes: Create a new `Axes` object containing the given collection without labels /styles. **CollectionAxes(Iterable)** - Constructor for class fern.tools.gnuplot.CollectionAxes: Create a new `Axes` object containing a collection (which is copied from the given iterable) without labels /styles. **CollectionAxes(Collection, String[], String[])** - Constructor for class fern.tools.gnuplot.CollectionAxes: Create a new `Axes` object containing the given collection with the given labels /styles (either can be `null`). **CollectionAxes(Iterable, String[], String[])** - Constructor for class fern.tools.gnuplot.CollectionAxes: Create a new `Axes` object containing a collection (which is copied from the given iterable) with the given labels /styles (either can be `null`). **ColorCalculator** - Class in fern.cytoscape: **ColorCalculator()** - Constructor for class fern.cytoscape.ColorCalculator: **colorCalculator** - Static variable in class fern.cytoscape.FernVisualStyle: **ColorCalculator.Scale** - Enum in fern.cytoscape: **ColorPicker** - Class in fern.cytoscape.ui: **ColorPicker(Frame)** - Constructor for class fern.cytoscape.ui.ColorPicker: **ColorSpectrum** - Class in fern.tools: Provides methods to assign colors to numbers between 0 and 1. **ColorSpectrum()** - Constructor for class fern.tools.ColorSpectrum: **commands** - Variable in class fern.tools.gnuplot.GnuPlot: **ComplexDependenciesPropensityCalculator** - Interface in fern.network: In a SBML network, the propensities of reaction are calculated by using the kineticLaw tag, which contains a MathML expression. **computePath(String, String...)** - Method in class fern.analysis.ShortestPath: Compute the shortest paths from some source species to one species. **computePath(NodeChecker, String, String...)** - Method in class fern.analysis.ShortestPath: Compute the shortest paths from some source species to one species by only using parts of the network specified by the `NodeChecker` `checker`. **computePaths(String...)** - Method in class fern.analysis.ShortestPath: Compute all shortest paths from some source species. **computePaths(NodeChecker, String...)** - Method in class fern.analysis.ShortestPath: Compute all shortest paths from some source species by only using parts of the network specified by the `NodeChecker` `checker`. **ConfigReader** - Class in fern.tools: Reads simple config files for test cases and makes the data of it accessible. **ConfigReader(String)** - Constructor for class fern.tools.ConfigReader: Reads the given file and stores the data. **containsNetworkAnnotation(String)** - Method in class fern.cytoscape.CytoscapeAnnotationManager: **containsNetworkAnnotation(String)** - Method in interface fern.network.AnnotationManager: Returns true iff the network contains an annotation of the specified type. **containsNetworkAnnotation(String)** - Method in class fern.network.AnnotationManagerImpl: **containsNetworkAnnotation(String)** - Method in class fern.network.fernml.FernMLAnnotationManager: **containsReactionAnnotation(int, String)** - Method in class fern.cytoscape.CytoscapeAnnotationManager: **containsReactionAnnotation(int, String)** - Method in interface fern.network.AnnotationManager: Returns true iff the reaction contains an annotation of the specified type. **containsReactionAnnotation(int, String)** - Method in class fern.network.AnnotationManagerImpl: **containsReactionAnnotation(int, String)** - Method in class fern.network.fernml.FernMLAnnotationManager: **containsSpeciesAnnotation(int, String)** - Method in class fern.cytoscape.CytoscapeAnnotationManager: **containsSpeciesAnnotation(int, String)** - Method in interface fern.network.AnnotationManager: Returns true iff the species contains an annotation of the specified type. **containsSpeciesAnnotation(int, String)** - Method in class fern.network.AnnotationManagerImpl: **containsSpeciesAnnotation(int, String)** - Method in class fern.network.fernml.FernMLAnnotationManager: **convertIntToDouble(int[])** - Static method in class fern.tools.NumberTools: Performs a deep cast of an int[]. **count** - Variable in class fern.benchmark.SimulatorPerformance: Contains the number of iterations done for using in the `present`-method of extending classes. **createAdjacencyLists()** - Method in class fern.cellDesigner.CellDesignerNetworkWrapper: **createAdjacencyLists()** - Method in class fern.cytoscape.CytoscapeNetworkWrapper: **createAdjacencyLists()** - Method in class fern.network.AbstractNetworkImpl: Reminds extending class to fill `AbstractNetworkImpl.adjListPro` and `AbstractNetworkImpl.adjListRea`. **createAdjacencyLists()** - Method in class fern.network.creation.AutocatalyticNetwork: Creates the adjacency lists for this network. **createAdjacencyLists()** - Method in class fern.network.fernml.FernMLNetwork: Creates the adjacency lists by parsing the jdom tree. **createAdjacencyLists()** - Method in class fern.network.sbml.SBMLNetwork: **createAmountManager()** - Method in class fern.cellDesigner.CellDesignerNetworkWrapper: **createAmountManager()** - Method in class fern.cytoscape.CytoscapeNetworkWrapper: **createAmountManager()** - Method in class fern.network.AbstractNetworkImpl: Reminds extending class to fill `AbstractNetworkImpl.amountManager`. **createAmountManager()** - Method in class fern.network.creation.AutocatalyticNetwork: Creates the `AmountManager` for this network. **createAmountManager()** - Method in class fern.network.fernml.FernMLNetwork: **createAmountManager()** - Method in class fern.network.sbml.SBMLNetwork: **createAnnotationManager()** - Method in class fern.cellDesigner.CellDesignerNetworkWrapper: **createAnnotationManager()** - Method in class fern.cytoscape.CytoscapeNetworkWrapper: **createAnnotationManager()** - Method in class fern.network.AbstractNetworkImpl: Reminds extending class to fill `AbstractNetworkImpl.annotationManager`. **createAnnotationManager()** - Method in class fern.network.creation.AutocatalyticNetwork: Creates the `AnnotationManager` for this network. **createAnnotationManager()** - Method in class fern.network.fernml.FernMLNetwork: Creates the `AnnotationManager` as a `FernMLAnnotationManager`. **createAnnotationManager()** - Method in class fern.network.sbml.SBMLNetwork: **createEventHandlers()** - Method in class fern.network.sbml.SBMLNetwork: **createHistogram()** - Method in class fern.example.HistogramDistanceTestSet: **createHistogram(Collection<double[]>, int, int...)** - Static method in class fern.tools.NumberTools: Creates a histogram from the given data. **createHistogram(int[])** - Static method in class fern.tools.NumberTools: Create a histogram for the given array. **createHistogramAsMap(int[])** - Static method in class fern.tools.NumberTools: Create a histogram map for the given array. **createInverse(int[])** - Static method in class fern.tools.NumberTools: Creates the inverse array to the given array. **createInverseAsMap(int[])** - Static method in class fern.tools.NumberTools: Creates the inverse map to the given array. **createPropensityCalulator()** - Method in class fern.cellDesigner.CellDesignerNetworkWrapper: **createPropensityCalulator()** - Method in class fern.cytoscape.CytoscapeNetworkWrapper: **createPropensityCalulator()** - Method in class fern.network.AbstractNetworkImpl: Reminds extending class to fill `AbstractNetworkImpl.propensitiyCalculator`. **createPropensityCalulator()** - Method in class fern.network.creation.AutocatalyticNetwork: Creates the `PropensityCalculator` for this network. **createPropensityCalulator()** - Method in class fern.network.fernml.FernMLNetwork: Does nothing, the `PropensityCalculator` is created in `createAdjacencyLists` because the reactions constants are already parsed there. **createPropensityCalulator()** - Method in class fern.network.sbml.SBMLNetwork: **createRandomDoubleArray(int, AbstractDistribution)** - Method in class fern.benchmark.Benchmark: Creates a test set containing `size` random numbers of the distribution `dist`. **createSpeciesAdjacencyLists()** - Method in class fern.analysis.AnalysisBase: Creates the adjacency lists for the molecule species. **createSpeciesMapping()** - Method in class fern.cellDesigner.CellDesignerNetworkWrapper: **createSpeciesMapping()** - Method in class fern.cytoscape.CytoscapeNetworkWrapper: **createSpeciesMapping()** - Method in class fern.network.AbstractNetworkImpl: Reminds extending class to fill `AbstractNetworkImpl.speciesIdToIndex` and `AbstractNetworkImpl.indexToSpeciesId`. **createSpeciesMapping()** - Method in class fern.network.creation.AutocatalyticNetwork: **createSpeciesMapping()** - Method in class fern.network.fernml.FernMLNetwork: Creates the species mapping by parsing the jdom tree. **createSpeciesMapping()** - Method in class fern.network.sbml.SBMLNetwork: **cumSum(int[])** - Static method in class fern.tools.NumberTools: Replaces the values in the given array with the cumulative sum of the array. **CytoscapeAnnotationManager** - Class in fern.cytoscape: **CytoscapeAnnotationManager(CytoscapeNetworkWrapper)** - Constructor for class fern.cytoscape.CytoscapeAnnotationManager: **CytoscapeColorChangeObserver** - Class in fern.cytoscape: **CytoscapeColorChangeObserver(boolean, boolean, Simulator, CytoscapeNetworkWrapper, FernVisualStyle, String[], double)** - Constructor for class fern.cytoscape.CytoscapeColorChangeObserver: **CytoscapeColorChangeObserver.ColorChangingNodeAppeareanceCalculator** - Class in fern.cytoscape: **CytoscapeColorChangeObserver.ColorChangingNodeAppeareanceCalculator(NodeAppearanceCalculator)** - Constructor for class fern.cytoscape.CytoscapeColorChangeObserver.ColorChangingNodeAppeareanceCalculator: **CytoscapeNetworkWrapper** - Class in fern.cytoscape: **CytoscapeNetworkWrapper(NetworkChecker, CyNetwork, CyNetworkView, CyAttributes)** - Constructor for class fern.cytoscape.CytoscapeNetworkWrapper: **CytoscapeVisualizer** - Class in fern.cytoscape: **CytoscapeVisualizer()** - Constructor for class fern.cytoscape.CytoscapeVisualizer: **CytoscapeVisualizer.ShowMainFrameAction** - Class in fern.cytoscape: **CytoscapeVisualizer.ShowMainFrameAction()** - Constructor for class fern.cytoscape.CytoscapeVisualizer.ShowMainFrameAction: **CytoscapeVisualizer.SimulationAction** - Class in fern.cytoscape: **CytoscapeVisualizer.SimulationAction()** - Constructor for class fern.cytoscape.CytoscapeVisualizer.SimulationAction

---


|  |  |  |
| --- | --- | --- |
| **Overview** \| Package \| Class \| Use \| **Tree** \| **Deprecated** \| **Index** \| **Help** | |  |
| **PREV LETTER**   **NEXT LETTER** | **FRAMES**    **NO FRAMES**     **All Classes** |  |


A B C D E F G H I J L M N O P R S T U V 

---
